# The Effectiveness of Varying Combination Ratios of *A*. *cordifolia* and *M*. *indica* against Field and Laboratory Strains of *P*. *falciparum In Vitro*

**DOI:** 10.1155/2020/8836771

**Published:** 2020-11-14

**Authors:** Yakubu Jibira, Elizabeth Cudjoe, Frederick M. Tei-Maya, Benjamin Ayensu, Linda E. Amoah

**Affiliations:** ^1^Department of Pharmacology, College of Health Science, Kwame Nkrumah University of Science and Technology, Kumasi, Ghana; ^2^Department of Biochemistry, Cell and Molecular Biology, Univeristy of Ghana, Accra, Ghana; ^3^Immunology Department, Noguchi Memorial Institute for Medical Research, University of Ghana, Accra, Ghana

## Abstract

**Background:**

Drug resistance in malaria is a global problem, with reports of *Plasmodium* parasites resistant to the current first-line antimalarial drug, artemisinin, expanding from Southeast Asia to Africa. There is therefore an urgent need to identify new drug candidates that will be effective against the existing malaria parasites. Drug combination therapy presents a myriad of advantages over monotherapy including delayed onset of resistance, potentiation, and synergism. This present study explored the effectiveness of combinations of aqueous extracts of *Alchornea cordifolia* (*A*. *cordifolia)* and *Mangifera indica* (*M*. *indica)* at clearing both laboratory and field isolates of *P*. *falciparum*.

**Methods:**

Synchronized ring stage cultures of field (FA08) and laboratory strains (NF54 and CamWT_C580Y) of *P*. *falciparum* were subjected to combinations of different concentrations and ratios of aqueous extracts of *A*. *cordifolia* and *M*. *indica.* The growth inhibition of the individual plant extracts and their combinatory effects were studied *in vitro* using SYBR Green I drug assay.

**Results:**

The *A*. *cordifolia* extract exhibited 50% inhibitory concentration (IC_50_) of 2.71, 7.80, and 3.56 *μ*g/mL against the NF54, CamWT_C580Y, and FA08 parasite strains, respectively. *Mangifera indica* exhibited IC_50_ of 18.11, 20.08, and 10.23 *μ*g/mL against the NF54, CamWT_C580Y, and FA08 parasite strains, respectively. Additive, synergistic and antagonistic interactions were observed at different combinations of *A*. *cordifolia* and *M*. *indica* extracts.

**Conclusion:**

A combination product containing *A. cordifolia* and *M*. *indica* has the potential to serve as an effective antimalarial as majority of the tested combinations of aqueous extracts of *A*. *cordifolia* and *M*. *indica* extracts exhibited synergistic effects in vitro against the NF54, CamWT_C580Y, and FA08 *P*. *falciparum* strains.

## 1. Background

Artemisinin combination therapy (ACT) is the first-line antimalarial drug recommended by the World Health Organization (WHO) for the treatment of uncomplicated malaria [1] and implemented by the National Malaria Control Program (NMCP) of malaria endemic countries including Ghana [2]. Although ACTs are highly effective against chloroquine- (CQ-) and sulphadoxine pyrimethamine- (SP-) resistant malaria parasites, they are generally much more expensive than the nonartemisinin antimalarials such as CQ and SP that were previously used to treat malaria [3]. In addition to the high cost, there are a large number of substandard (containing subtherapeutic doses) and counterfeit drugs that do not meet the designated quality standards and or specifications including ACTs present in the Sub-Saharan African market [4] that has resulted in a reduced confidence and purchase of the ACTs for malaria treatment. Herbal medicines have been used for malaria treatment since the ancient times [5] and were the basis for the discovery of some very potent antimalarial drugs such as quinine and artemisinin [6]. Herbal products are rapidly gaining popularity due to their natural organic origin and minimal known side effects [7]. Traditional herbal remedies have been adopted in treating malaria patients since ancient times, with over 1200 plant species from 160 families used for the traditional treatment of malaria [6, 8].

*Mangifera indica* (*M*. *indica*) is a common plant component of local herbal medicines on the Ghanaian market [9]. *Mangifera* spp. is a large genus of evergreen trees, distributed in tropical and subtropical parts of Southeast Asia. The popularity of the plant stems from its many medicinal attributes such as antiviral, antibacterial, and antiplasmodial [7, 10, 11]. Although there is a report of the antimalarial properties of mangiferin, a major component of *M*. *indica* [12], the active antimalarial components of *M*. *indica* have not been characterized and activity-guided bioassays are needed to identify the active compounds [13].

*Alchornea cordifolia* (*A*. *cordifolia*) is a West African plant that belongs to the family of *Euphorbiacea* [14]. The active compound in the leaves of *A*. *cordifolia* has been found to be ellagic acid, which is able to inhibit the growth of *P*. *falciparum* without any cytotoxicity [15].

Herbal antimalarial products, which are the most popular herbal products on the Ghanaian market, are usually composed of a mixture of a variety of herbal extracts [16]. Although the independent aqueous extracts of *A*. *cordifolia* and *M*. *indica* exhibit very good antimalarial activity against *P*. *falciparum* parasites [11, 17–19], knowledge on the antimalarial activity of different combinations of these two extracts is lacking. In this study, we identify the effects of different combinations of *A*. *cordifolia* and *M*. *indica* on laboratory and field isolates of *P*. *falciparum.*

## 2. Methods

### 2.1. Identification and Preparation of Herbal Extracts

The herbal extracts used in this study have previously been described [12]. Briefly, fresh leaves of *A*. *cordifolia* were obtained from Apooh in the Shama Ahanta East district of the Western Region (4° 54′ 26.6405^″^ N, 1° 49′ 6.6002^″^ W) and *M*. *indica,* from Adenta (5° 42′ 32.8931^″^ N, 0° 10′ 13.2384^″^ W) in the Adentan Municipality of the Greater Accra region of Ghana. The leaves were identified at the University of Ghana herbarium, Accra, and the herbarium at the Centre for Plant Medicine Research (CPMR), Mampong. The leaves were then air dried and ground into powder using a blender. Each set of ground leaves (21.5 g of *A*. *cordifolia* and 32 g of *M*. *indica*) were boiled in 450 mL of distilled water at 100°C for an hour. The solutions were left to cool at room temperature for 18 hours and then filtered using Whatman™ 54 filter paper. The filtered solutions were finally lyophilized using a Labconco™ Freeze Dryer. Stock concentrations of 50 mg/mL were prepared for both extracts by dissolving 500 mg of the lyophilized extract in 10 mL distilled water. The stock solutions were subsequently filtered through a 0.2 *μ*m Acodisc™ syringe filter and used immediately or stored at -20°C.

### 2.2. Culturing of Plasmodium Parasites

Asexual cultures of NF54 (MRA-1000: chloroquine sensitive), CamWT_C580Y (MRA-1250: artemisinin sensitive), and FA08 (Ghanaian culture adapted field isolate) were maintained *in vitro* using a modified method of Mustofa [17] and similar to Amoah et al. [20]. Briefly, the parasites were individually cultured at 4% hematocrit (O+ red blood cells (RBCs)) in complete parasite media (CPM: RPMI 1640 supplemented with 25 mM HEPES, 2 mM L-glutamine, 24.1 mM NaHCO_3_, 11.1 mM glucose, 50 *μ*g/mL gentamycin, and 0.5% Albumax II) in a T75 culture flask. The cultures were maintained in an incubator set at 37°C with daily media change with CPM and exchange of gas (92.5% nitrogen, 5.5% carbon dioxide, and 2% oxygen).

Synchronized ring stage parasites were obtained by treating a culture containing more than 5% ring stage parasites with a solution of 5% sorbitol for 10 minutes at room temperature. Two days (48 hours) after synchronization, the cultures, which were predominantly ring stage parasites were plated at 2% for the SYBR Green 1 assay.

### 2.3. SYBR Green I Asexual Parasite Drug Assay

A protocol similar to that described by Cudjoe et al. [11] and Smilkstein et al. [21] with some revisions was used to determine the inhibitory effects of the aqueous extracts of *A*. *cordifolia* and *M*. *indica* on different *P*. *falciparum* parasites. A schematic of plate was set up in Additional file 1. Briefly, 25 *μ*L of a fixed concentration (40, 20, 10, 5, and 0 *μ*g/mL) of *A*. *cordifolia* and another 25 *μ*L of a fixed concentration (100, 40, 20, 10, and 0 *μ*g/mL) of *M*. *indica* were dispensed in triplicate into the wells of a 96-well tissue culture plate (Additional file S1). The aqueous extracts of *A*. *cordifolia* and *M*. *indica* used in this study have been previously described [11]. Positive control wells were filled with 50 *μ*L of different concentrations (400–1 ng/mL) of the reference drug artesunate (AS) (Additional file S1). Two plates containing different mixtures of *A*. *cordifolia* and *M*. *indica* extracts and AS were set up for each parasite strain. A series of untreated infected RBCs set at 1%-0.25% parasitaemia was also set up in triplicates to serve as negative controls for the assay (Additional file S1). Each of the remaining wells were then supplemented with 50 *μ*L of ring stage-infected RBC (iRBCs) set at 1% parasitemia (either NF54, FA8, or CamWT_C580Y) and 4% hematocrit in CPM. The plates were then placed into a modular incubating chamber, gassed for 6 minutes and then incubated for 72 hours at 37°C. Two technical replicate plates were set up for each assay.

The plates were subsequently wrapped in aluminum foil and frozen overnight at -20°C. The plates were thawed at room temperature, and each well was then filled with 100 *μ*L of buffered SYBR Green (2x SYBR Green 1 dye in 20 mM Tris-HCl, pH 7.5 supplemented with 5 mM EDTA, 0.08% Triton X-100, and 0.008% saponin in PBS). The plate was wrapped again in aluminum foil, incubated at 37°C for 1 hour, and fluorescence was then read on a microplate reader at 497 nm excitation and 530 nm emission.

### 2.4. Statistical Analysis

For the SYBR Green 1 drug assays, the data obtained from the herbal extract-treated uninfected RBC was used as the background and subtracted from the corresponding infected RBC data set.

Data was converted into % inhibition using the formula:

(1) $$\begin{array}{c} \%\text{Inhibition}=100\times \left[1-\left(X-\min\right)/\left(\mathrm{Max}-\min\right)\right], \end{array}$$

where *X* is the signal at a given concentration of the inhibitor, min is the signal with 100% inhibition, and Max is the signal with no inhibition.

### 2.5. Isobologram analysis

The growth inhibition caused by *A*. *cordifolia*, and *M*. *indica* extracts and AS were individually normalized as percentages and plotted against the log concentration of the drugs. The data obtained was analysed using Compusyn (Compusyn Software, Combosyn, Inc., PD Science LLC, USA). The software is based on the Chou-Talalay method for drug combination (based on median-effect equation) which provides combination index- (CI-) isobologram equation that gives quantitative determination of drug interaction, where CI < 1, CI > 1, and CI = 1 denotes synergism, antagonism, and additive effect, respectively [22]. The resulting sigmoidal dose response curves used to calculate the 50% inhibitory concentration (IC_50_) [22, 23]. Dose-response curves were also obtained and analyzed after the coadministration of *A*. *cordifolia* and *M*. *indica* extracts in fixed combination ratios. For each combination, the IC_50_ (experimental), CI, and its associated fraction affected (Fa) were evaluated by a quantitative diagnostic plot (Fa-Cl) analysis of the log dose-response curve obtained using the three formulas below:

(2) $$\begin{array}{c} \frac{\text{Fa}}{\text{Fu}}={\left[\frac{D}{\text{Dm}}\right]}^{m},\\ \log\left[\frac{\text{Fa}}{\text{Fu}}\right]=m\left[\log D\right]-m\left[\log\text{Dm}\right],\\ \text{CI}=\overset{n}{\underset{j=1}{\sum }}\left[\frac{D}{\text{Dx}}\right], \end{array}$$

where Fa is the fraction affected, Fu is the fraction unaffected, *D* is the dose required to produce Fa, Dm is the the median dose effect (IC_50_); *m* is the dynamic order (sigmoidicity), and Dx is the7 the dose of each drug alone that exerts *X* % inhibition.

*P* values for statistical significance were set at 0.05.

## 3. Results

**Figure 1:**
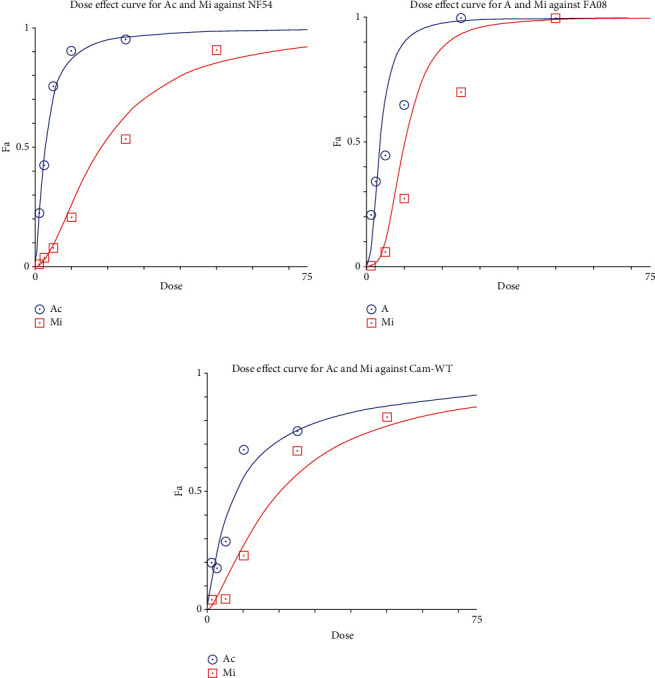
Dose-response curves for *A*. *cordifolia* (Ac) and *M*. *indica* (Mi) against *P*. *falciparum* parasites. The SYBR Green I in vitro drug assay was used to determine the dose-response curves for the NF54, FA08, and Cam-WT (CamWT_C580Y) parasite strains.

The aqueous extract of *A*. *cordifolia* primarily displayed a sigmoid-shaped dose-response relationship (Figure 1) with an approximate IC_50_ against NF54, CamWT_C580Y, and FA08 *Plasmodium* parasite strain of 2.71, 7.80, and 3.56 *μ*g/mL (Table 1). The IC_50_ values for *M*. *indica* were 18.11, 20.08, and 10.23 *μ*g/mL against the NF54, CamWT_C580Y, and FA08 *Plasmodium* parasite strains, respectively, were lower than those obtained for *A*. *cordifolia.*

The activity of *A. cordifolia* was relatively similar amongst the three parasite strains but was highest against the NF54 strain, whilst the activity of *M*. *indica* was highest in the FA08 strain (Table 1, Additional file Figure S2).

Taking cognizance of the drug-interaction assay, the combinations of *A*. *cordifolia* with *M*. *indica* were synergistic, antagonistic, and additive at different combinations ratios (Table 1). The activity of combinations of *A*. *cordifolia* with *M*. *indica* against the CamWT_C580Y and NF54 strains showed CI of less than 1 suggesting synergy except for ratios of when the combinations were at a 10 : 1 and 20 : 1 ratio (NF54) or 10 : 1 and 20 : 1 against the CamWT_C580Y parasites. The degree of synergism was stronger at a 1 : 4 ratio of *A*. *cordifolia* with *M*. *indica* (CI = 0.167) followed by the 1 : 2 ratio (CI = 0.192) and finally the 2 : 1 (CI = 0.300) against CamWT_C580Y parasites (Table 1).

A significant suppression of parasite growth was observed when the NF54 parasite strain was treated with 19.67 *μ*g/mL of *A*. *cordifolia* and 98.98 *μ*g/mL for *M*. *indica.* Similarly, a significant suppression in the growth of the FA8 strain was observed when treated with 27.62 *μ*g/mL of *A*. *cordifolia* and 13.73 *μ*g/mL *M*. *indica*. A significant reduction in the growth of the CamWT_580Y parasite strain was observed when the parasites were treated with 150.65 *μ*g/mL of *A*. *cordifolia* and 169.48 *μ*g/mL *M*. *indica* (Table 2). In addition, combinations of *A*. *cordifolia* with *M*. *indica* at 2 : 1, 1 : 1, and 1 : 2 ratios showed synergistic effects against the NF54 and CamWT_580Y parasites and antagonistic effects at ratios of 2 : 1, 1 : 1, 1 : 2, and 10 : 1 against NF54, FA8, and CamWT_580Y. The combination caused more significant (*P* < 0.05) suppression in parasitaemia burden with estimated dose of 20.07 *μ*g/mL against NF54, 44.28 *μ*g/mL against FA8, and 31.26 *μ*g/mL against CamWT_580Y at 1 : 2, 1 : 1, and 2 : 1 ratios, respectively.

## 4. Discussion

Sustainable malaria control requires a combination of interventions and tools as well as research and development of enhanced strategies including vaccines, drugs, diagnostics, and vector management approaches. Antimalarial drugs such as CQ, SP, quinine, and recently the artemisinins have played a vital role in malaria control globally [24]. These drugs mainly target the erythrocytic phase of the infection, which is the phase of infection that presents signs and symptoms [25].

The rapid development of resistance to these commonly used antimalarial drugs by the malaria parasite has led to the recommendation that drug therapy should target multiple targets, so as to reduce the development of resistance. Suggesting the need for the use of drug combinations or a single drug that has multiple targets. There is also a major challenge existing in the supply and use of antimalarial drug combination therapies, particularly in Africa. The antimalarial activities of aqueous extracts of *A*. *cordifolia* and *M*. *indica* have recently been confirmed against a number of *P*. *falciparum* isolates [11]. This present study goes further to investigate the antimalarial activity of combinations of aqueous extracts of *A*. *cordifolia* and *M*. *indica in vitro*.

The aqueous extracts of both *A*. *cordifolia* and *M*. *indica* clearly suppressed the growth of all the three isolates of *P*. *falciparum* used in the study. This supports previous reports of extracts of both *A*. *cordifolia* and *M*. *indica* exhibiting antimalarial activity and also supports the traditional use of various parts of *A*. *cordifolia* and *M*. *indica* in the treatment of malaria [18, 26, 27]. The IC_50_ values reported for NF54 and CamWT_C580Y are similar to a recent report that used a slightly different plate set up for the sybrgreen 1 assay [11]. There was only one tested ratio of *A*. *cordifolia* and *M*. *indica* that exhibited synergistic effects against all the three parasite strains and also one tested ratio that yielded antagonistic effects against all the three parasite strains tested. This demonstrates that extensive studies using multiple parasite strains are required to identify the most appropriate combination ratio for *A*. *cordifolia* and *M*. *indica* as it is possible that a combination ratio that exerts high levels of synergistic effects on a tested parasite strain can be antagonistic against a different parasite strain. The observation that different combination ratios can result in different effects support previous reports where different amounts of cepharanthine combined with atovaquone and lumefantrine resulted in synergistic and additive effects against the W2 strain of *P*. *falciparum* [25].

The mode of action of *A*. *cordifolia* against *P*. *falciparum* is likely different from that of *M*. *indica* as the activity of *A*. *cordifolia* was highest against the NF54 strain but the activity of *M*. *indica* was highest in the FA08 strain (Table 1). The possibility of these two extracts having different modes of action against the malaria parasite make them ideal combination partners for the treatment of malaria. The *in vitro* antimalarial interactions of *A*. *cordifolia* in combination with *M*. *indica* had less than 1 combination index (CI) against CamWT_580Y depicting a strong synergy. Also, values obtained with five *A*. *cordifolia* combinations indicate a synergistic interaction of the *M*. *indica* against the NF54 strain. Artemisinin-based combination therapy (ACT) is now the mainstay in the treatment of malaria in Africa due it efficacy in the rapid clearance of symptoms and parasites and profound efficacy and low probability of drug-resistance development [24, 28].

This present study focused on the combination of aqueous extracts of *A*. *cordifolia* and *M*. *indica*, with the aim of developing novel combinational therapies to preempt the advancement of resistance to existing antimalarial drugs. As with ACT treatment, crude herbal extracts and their mixtures are expected to delay the onset of antimalarial drug resistance relative to single-component therapies due to the likelihood of the variant constituent components acting on different drug targets within the parasite [29]. Although the actual mechanism of action of either *A*. *cordifolia* or *M*. *indica* as antimalarial is unknown, the fact that growth inhibition of different parasite strains was effective suggests that appropriate combinations of *A*. *cordifolia* and *M*. *indica* have the potential to be used as potent schizonticides against a variety of *P*. *falciparum* parasites.

## 5. Conclusions

A combination product containing *A*. *cordifolia* and *M*. *indica* has the potential to serve as an effective antimalarial as majority of the tested combinations of aqueous extracts of *A*. *cordifolia* and *M*. *indica* extracts exhibited synergistic effects in vitro against the NF54, CamWT_C580Y, and FA08 *P*. *falciparum* strains.

## Figures and Tables

**Table 1:** The drug **c**ombination of *A*. *cordifolia* with *M*. *indica* against NF54, FA08, and CamWT_C580Y *Plasmodium* parasite strains.

Drugs	HE ratio	IC_50_	CI	Interaction	Correlation coefficient (*r*)
			(Fa = 95)		
*NF54*					
Ac	N/A	2.71	N/A	N/A	0.988
Mi	N/A	18.11	N/A	N/A	0.988
Ac + Mi	02 : 01	4.98	0.75	Syn	0.999
Ac + Mi	01 : 02	8.58	0.58	Syn	1
Ac + Mi	01 : 01	6.43	0.78	Syn	1
Ac + Mi	04 : 01	4.52	0.72	Syn	1
Ac + Mi	01 : 04	10.04	0.66	Syn	1
Ac + Mi	01 : 05	10.54	0.61	Syn	1
Ac + Mi	10 : 01	13.60	2.56	Anta	0.995
Ac + Mi	20 : 01	16.40	2.83	Anta	1
*FA08*					
Ac	N/A	3.55	N/A	N/A	0.888
Mi	N/A	10.23	N/A	N/A	0.934
Ac + Mi	02 : 01	770.26	3.348*E*8	Anta	0.134
Ac + Mi	01 : 02	10.92	3.03	Anta	0.786
Ac + Mi	01 : 01	11.99	2.41	Anta	0.89
Ac + Mi	04 : 01	20.73	15.34	Anta	1
Ac + Mi	01 : 04	10.74	0.90	Syn	1
Ac + Mi	10 : 01	19.81	2.65	Anta	1
Ac + Mi	20 : 01	22.33	2.29	Anta	1
*CamWT*					
Ac	N/A	7.80	N/A	N/A	0.933
Mi	N/A	20.08	N/A	N/A	0.938
Ac + Mi	02 : 01	13.47	0.30	Syn	1
Ac + Mi	01 : 02	11.38	0.19	Syn	1
Ac + Mi	01 : 01	12.93	0.32	Syn	1
Ac + Mi	04 : 01	17.42	0.79	Syn	1
Ac + Mi	01 : 04	10.18	0.17	Syn	1
Ac + Mi	01 : 05	13.72	0.55	Syn	1
Ac + Mi	10 : 01	14.57	1.05	Add	0.976
Ac + Mi	20 : 01	4.91	6.75	Anta	1

IC_50_ (*μ*g/mL) signifies potency, and *r* represents linear correlation coefficient. CI = 1, <1, and >1 depict additive effect, synergism, and antagonism, respectively. The dose and inhibition effect data were obtained from the SYBR Green I asexual Parasite drug assay in-vitro (*n* = 3). The data in this table was generated from the Compusyn software report. Ac: *A*. *cordifolia*; Mi: *M*. *indica* extract; CamWT: CamWT_580Y; Syn: synergy; Add: additive; anta: antagonistic.

**Table 2:** The Compusyn drug combination estimated dose of *A*. *cordifolia* with *M*. *indica* on the three parasite strains.

	HE/combo	CI value	Dose Ac	Dose Mi
NF54	Ac		19.67	
	Mi			98.98
	A2 : M1	0.75	13.38	6.69
	A1 : M1	0.78	12.81	12.81
	A10 : M1	2.56	49.41	4.94

FA8	Ac		13.73	
	Mi			27.62
	A2 : M1	3.348*E*8	3.681*E*9	1.840*E*9
	A1 : M2	3.03	20.85	41.71
	A1 : M1	2.41	22.14	22.14

CamWT	Ac		150.65	
	Mi			169.48
	A1 : M2	0.19	10.42	20.84
	A1 : M1	0.32	25.44	25.44
	A10 : M1	1.05	145.40	14.54

CI = 1, <1, and >1 depict additive effect, synergism, and antagonism, respectively. Ac: *A*. *cordifolia*; Mi: *M*. *indica* extract; CamWT: CamWT_580Y; syn: synergy; add: additive; anta: antagonistic.

## Data Availability

The data used to support the findings of this study are included within the article.

## Supplementary Materials

Supplementary Materials Additional file Figure S1: drug assay plate set up. Additional file Figure S2: graphical representation of combination growth inhibition data.
