# Supplementary material for: The Effectiveness of Varying Combination Ratios of A. cordifolia and M. indica against Field and Laboratory Strains of P. falciparum In Vitro
Source: J Parasitol Res. 2020 Nov 14;2020:8836771. doi: 10.1155/2020/8836771 (PMC7691008; doi:10.1155/2020/8836771)
Supplement: Supplementary Materials — Additional file Figure S1: drug assay plate set up. Additional file Figure S2: graphical representation of combination growth inhibition data. [file 8836771.f1.pptx]

## Slide 1
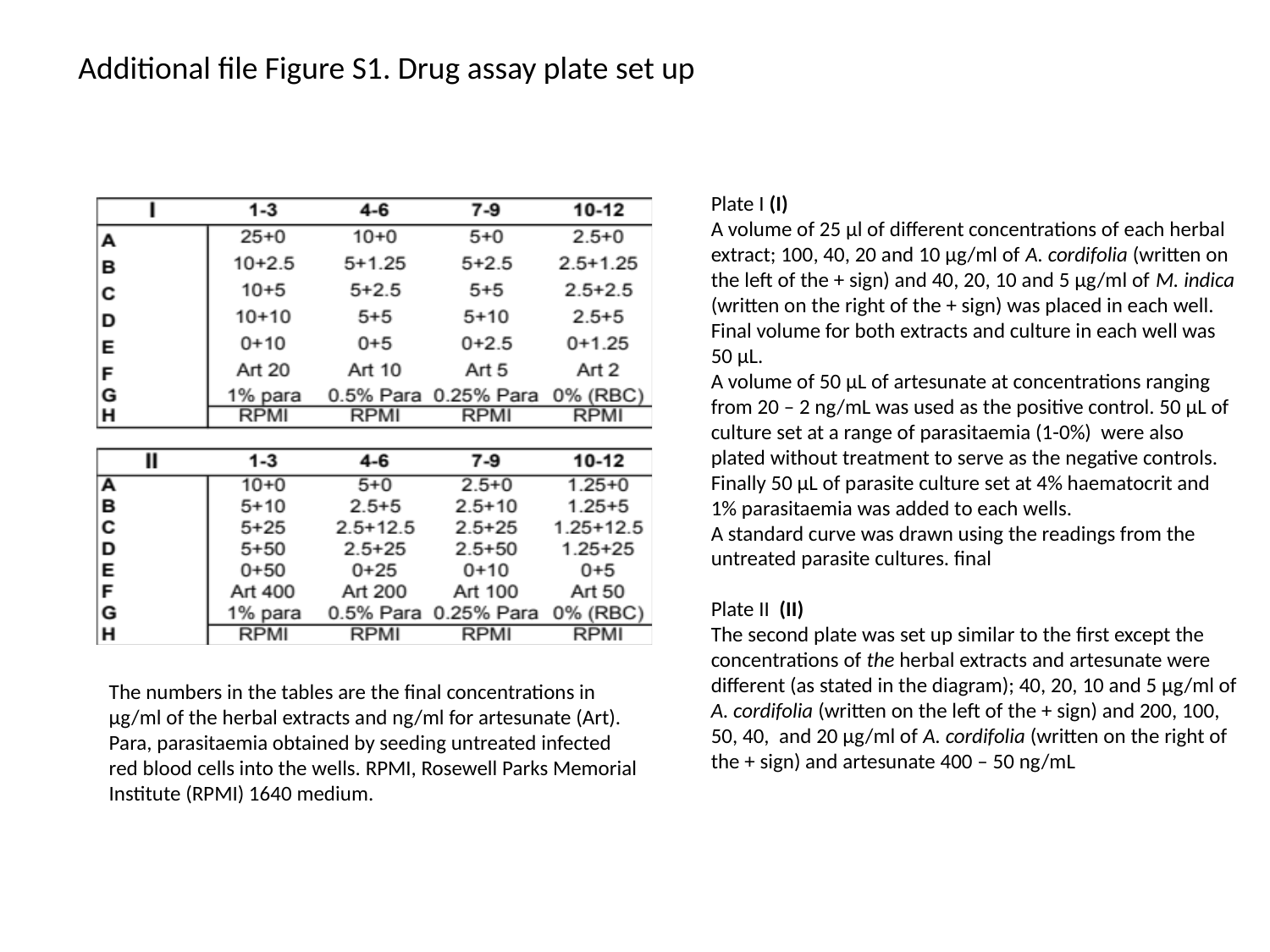

Additional file Figure S1. Drug assay plate set up
Plate I (I)
A volume of 25 μl of different concentrations of each herbal extract; 100, 40, 20 and 10 μg/ml of A. cordifolia (written on the left of the + sign) and 40, 20, 10 and 5 μg/ml of M. indica (written on the right of the + sign) was placed in each well. Final volume for both extracts and culture in each well was 50 µL.
A volume of 50 µL of artesunate at concentrations ranging from 20 – 2 ng/mL was used as the positive control. 50 µL of culture set at a range of parasitaemia (1-0%) were also plated without treatment to serve as the negative controls. Finally 50 µL of parasite culture set at 4% haematocrit and 1% parasitaemia was added to each wells.
A standard curve was drawn using the readings from the untreated parasite cultures. final
Plate II (II)
The second plate was set up similar to the first except the concentrations of the herbal extracts and artesunate were different (as stated in the diagram); 40, 20, 10 and 5 μg/ml of A. cordifolia (written on the left of the + sign) and 200, 100, 50, 40, and 20 μg/ml of A. cordifolia (written on the right of the + sign) and artesunate 400 – 50 ng/mL
The numbers in the tables are the final concentrations in μg/ml of the herbal extracts and ng/ml for artesunate (Art). Para, parasitaemia obtained by seeding untreated infected red blood cells into the wells. RPMI, Rosewell Parks Memorial Institute (RPMI) 1640 medium.

## Slide 2
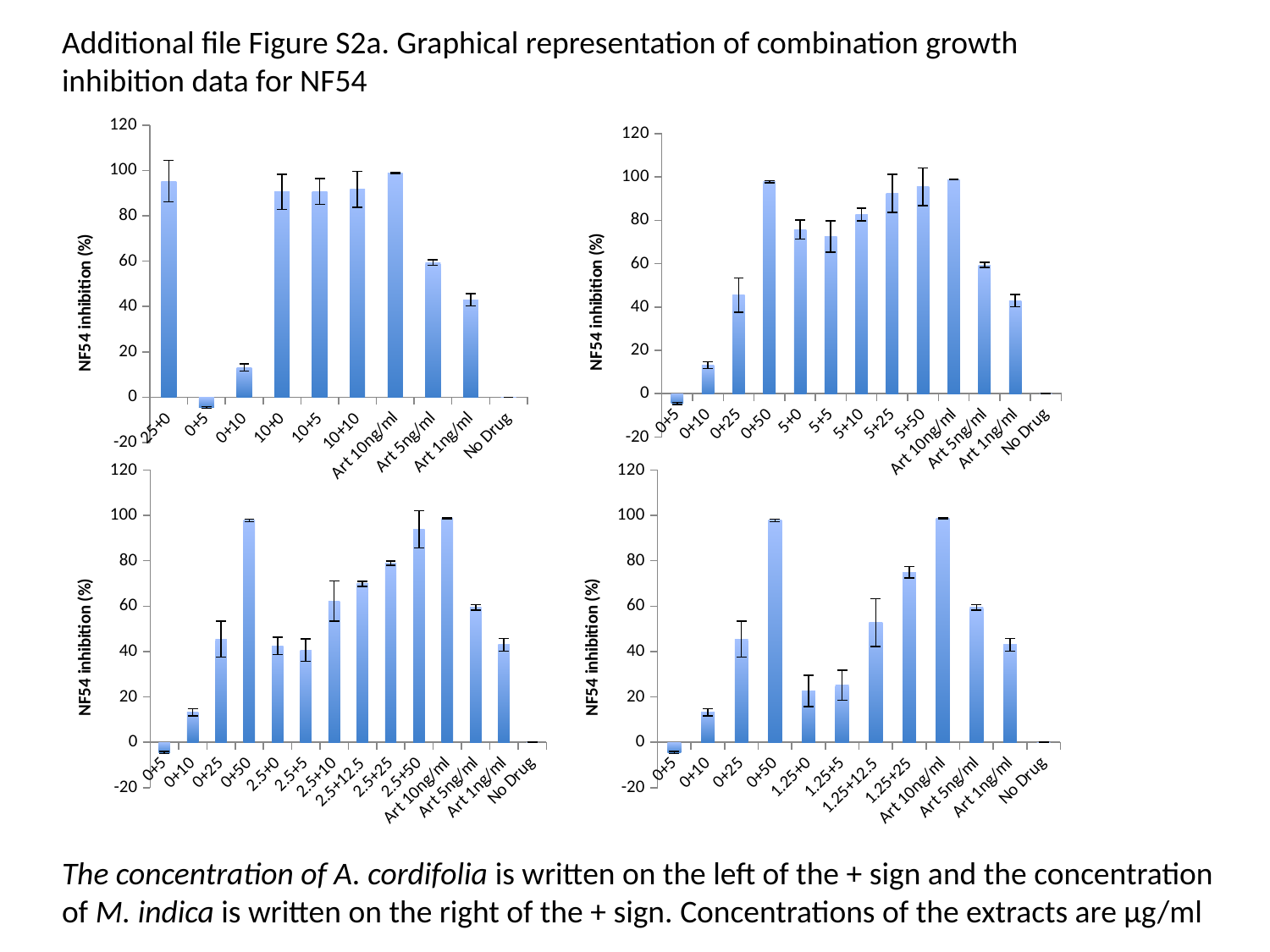

Additional file Figure S2a. Graphical representation of combination growth inhibition data for NF54
### Chart
| Category | 13.16246448 |
|---|---|
| 25+0 | 95.25585657311451 |
| 0+5 | -4.474097905760432 |
| 0+10 | 13.16246447950703 |
| 10+0 | 90.61893359451159 |
| 10+5 | 90.76404484744931 |
| 10+10 | 91.66616595441495 |
| Art 10ng/ml | 98.81285852011553 |
| Art 5ng/ml | 59.43274381536564 |
| Art 1ng/ml | 42.98106383961179 |
| No Drug | -4.518140739318e-06 |
### Chart
| Category | |
|---|---|
| 0+5 | -4.474097905760432 |
| 0+10 | 13.16246447950703 |
| 0+25 | 45.43893240310237 |
| 0+50 | 97.84484686620401 |
| 5+0 | 75.79213567852328 |
| 5+5 | 72.4878853988698 |
| 5+10 | 82.68376533558563 |
| 5+25 | 92.42829197434662 |
| 5+50 | 95.4505654130921 |
| Art 10ng/ml | 98.81285852011553 |
| Art 5ng/ml | 59.43274381536564 |
| Art 1ng/ml | 42.98106383961179 |
| No Drug | -4.518140739318e-06 |
### Chart
| Category | |
|---|---|
| 0+5 | -4.474097905760432 |
| 0+10 | 13.16246447950703 |
| 0+25 | 45.43893240310237 |
| 0+50 | 97.84484686620401 |
| 2.5+0 | 42.45575510733573 |
| 2.5+5 | 40.69121766997328 |
| 2.5+10 | 62.27057780340397 |
| 2.5+12.5 | 69.8902666687727 |
| 2.5+25 | 79.02642221079282 |
| 2.5+50 | 93.81937995905571 |
| Art 10ng/ml | 98.81285852011553 |
| Art 5ng/ml | 59.43274381536564 |
| Art 1ng/ml | 42.98106383961179 |
| No Drug | -4.518140739318e-06 |
### Chart
| Category | |
|---|---|
| 0+5 | -4.474097905760432 |
| 0+10 | 13.16246447950703 |
| 0+25 | 45.43893240310237 |
| 0+50 | 97.84484686620401 |
| 1.25+0 | 22.6201943952007 |
| 1.25+5 | 25.150642052669 |
| 1.25+12.5 | 52.6892456105052 |
| 1.25+25 | 75.018629703172 |
| Art 10ng/ml | 98.81285852011553 |
| Art 5ng/ml | 59.43274381536564 |
| Art 1ng/ml | 42.98106383961179 |
| No Drug | -4.518140739318e-06 |The concentration of A. cordifolia is written on the left of the + sign and the concentration of M. indica is written on the right of the + sign. Concentrations of the extracts are μg/ml

## Slide 3
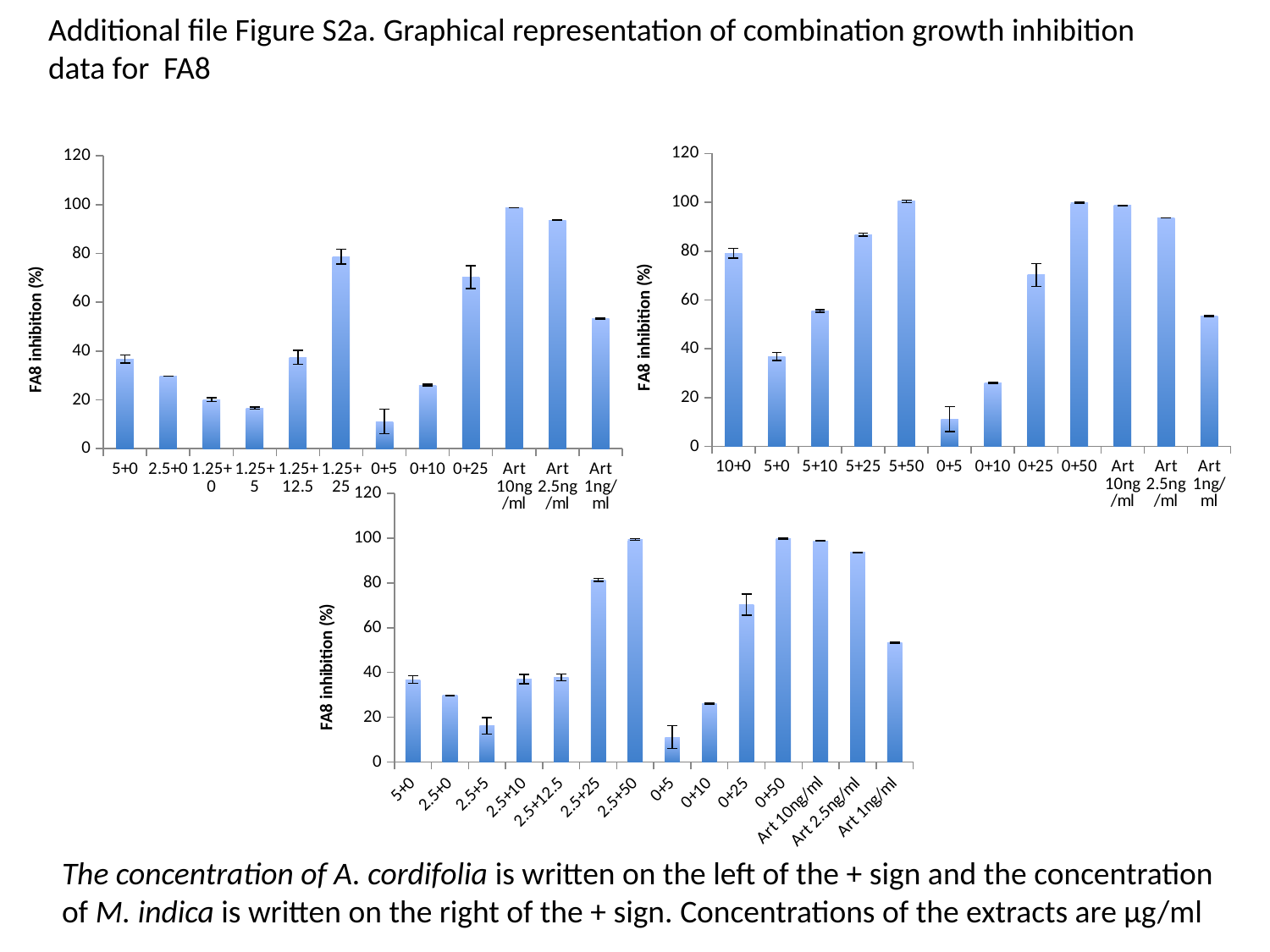

Additional file Figure S2a. Graphical representation of combination growth inhibition data for FA8
### Chart
| Category | |
|---|---|
| 10+0 | 79.13181666109065 |
| 5+0 | 36.80578788892605 |
| 5+10 | 55.4909668785547 |
| 5+25 | 86.81833389093339 |
| 5+50 | 100.4014720642355 |
| 0+5 | 11.1520017843203 |
| 0+10 | 26.05386416861826 |
| 0+25 | 70.23809523809526 |
| 0+50 | 99.78671796587489 |
| Art 10ng/ml | 98.73285379725661 |
| Art 2.5ng/ml | 93.60990297758443 |
| Art 1ng/ml | 53.33723653395785 |
### Chart
| Category | |
|---|---|
| 5+0 | 36.80578788892605 |
| 2.5+0 | 29.73402475744396 |
| 1.25+0 | 20.11542321846771 |
| 1.25+5 | 16.60254265640683 |
| 1.25+12.5 | 37.49163599866177 |
| 1.25+25 | 78.72476859596298 |
| 0+5 | 11.1520017843203 |
| 0+10 | 26.05386416861826 |
| 0+25 | 70.23809523809526 |
| Art 10ng/ml | 98.73285379725661 |
| Art 2.5ng/ml | 93.60990297758443 |
| Art 1ng/ml | 53.33723653395785 |
### Chart
| Category | |
|---|---|
| 5+0 | 36.80578788892605 |
| 2.5+0 | 29.73402475744396 |
| 2.5+5 | 16.18434258949481 |
| 2.5+10 | 37.0399799263968 |
| 2.5+12.5 | 37.8094680495149 |
| 2.5+25 | 81.34548901527825 |
| 2.5+50 | 99.37688190030107 |
| 0+5 | 11.1520017843203 |
| 0+10 | 26.05386416861826 |
| 0+25 | 70.23809523809526 |
| 0+50 | 99.78671796587489 |
| Art 10ng/ml | 98.73285379725661 |
| Art 2.5ng/ml | 93.60990297758443 |
| Art 1ng/ml | 53.33723653395785 |The concentration of A. cordifolia is written on the left of the + sign and the concentration of M. indica is written on the right of the + sign. Concentrations of the extracts are μg/ml

## Slide 4
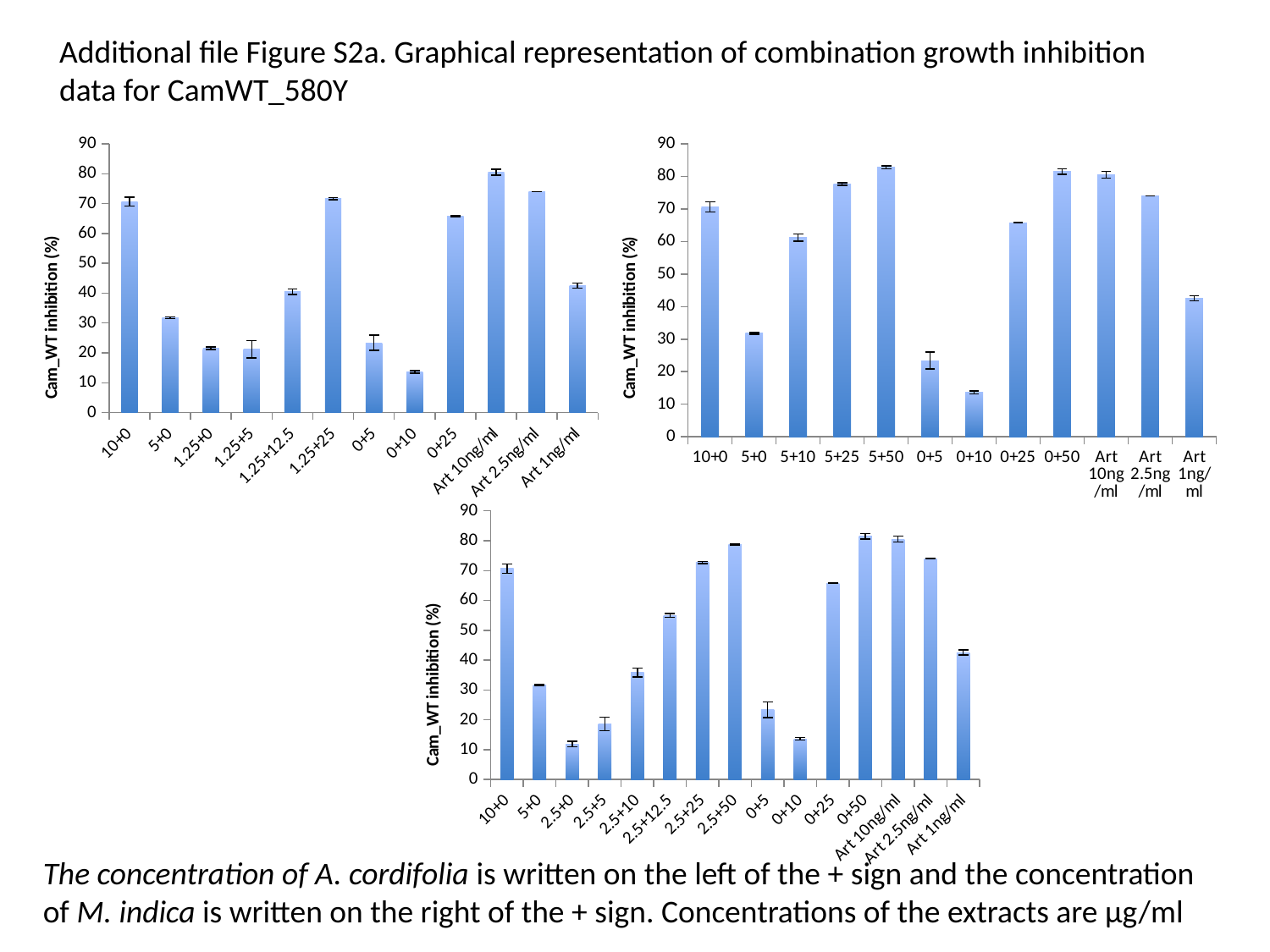

Additional file Figure S2a. Graphical representation of combination growth inhibition data for CamWT_580Y
### Chart
| Category | |
|---|---|
| 10+0 | 70.6613226452906 |
| 5+0 | 31.75851703406812 |
| 1.25+0 | 21.51803607214428 |
| 1.25+5 | 21.2374749498998 |
| 1.25+12.5 | 40.52104208416833 |
| 1.25+25 | 71.62992651970605 |
| 0+5 | 23.40180360721443 |
| 0+10 | 13.62725450901804 |
| 0+25 | 65.78657314629258 |
| Art 10ng/ml | 80.53607214428854 |
| Art 2.5ng/ml | 74.0230460921843 |
| Art 1ng/ml | 42.5751503006012 |
### Chart
| Category | |
|---|---|
| 10+0 | 70.6613226452906 |
| 5+0 | 31.75851703406812 |
| 5+10 | 61.22244488977956 |
| 5+25 | 77.66533066132264 |
| 5+50 | 82.84068136272545 |
| 0+5 | 23.40180360721443 |
| 0+10 | 13.62725450901804 |
| 0+25 | 65.78657314629258 |
| 0+50 | 81.4929859719439 |
| Art 10ng/ml | 80.53607214428854 |
| Art 2.5ng/ml | 74.0230460921843 |
| Art 1ng/ml | 42.5751503006012 |
### Chart
| Category | |
|---|---|
| 10+0 | 70.6613226452906 |
| 5+0 | 31.75851703406812 |
| 2.5+0 | 11.87374749498998 |
| 2.5+5 | 18.62725450901804 |
| 2.5+10 | 35.85170340681359 |
| 2.5+12.5 | 55.00501002004007 |
| 2.5+25 | 72.64529058116231 |
| 2.5+50 | 78.65397461589845 |
| 0+5 | 23.40180360721443 |
| 0+10 | 13.62725450901804 |
| 0+25 | 65.78657314629258 |
| 0+50 | 81.4929859719439 |
| Art 10ng/ml | 80.53607214428854 |
| Art 2.5ng/ml | 74.0230460921843 |
| Art 1ng/ml | 42.5751503006012 |The concentration of A. cordifolia is written on the left of the + sign and the concentration of M. indica is written on the right of the + sign. Concentrations of the extracts are μg/ml
